# Counting Activities Using Weakly Labeled Raw Acceleration Data: A Variable-Length Sequence Approach with Deep Learning to Maintain Event Duration Flexibility

**DOI:** 10.3390/s23115057

**Published:** 2023-05-25

**Authors:** Georgios Sopidis, Michael Haslgrübler, Alois Ferscha

**Affiliations:** 1 Pro2Future GmbH, Altenberger Strasse 69, 4040 Linz, Austria; michael.haslgruebler@pro2future.at; 2 Institute of Pervasive Computing, Johannes Kepler University, Altenberger Straße 69, 4040 Linz, Austria; ferscha@soft.uni-linz.ac.at

**Keywords:** artificial intelligence, deep learning, counting, weakly labeled data, variable length size, non-uniform shape data

## Abstract

This paper presents a novel approach for counting hand-performed activities using deep learning and inertial measurement units (IMUs). The particular challenge in this task is finding the correct window size for capturing activities with different durations. Traditionally, fixed window sizes have been used, which occasionally result in incorrectly represented activities. To address this limitation, we propose segmenting the time series data into variable-length sequences using ragged tensors to store and process the data. Additionally, our approach utilizes weakly labeled data to simplify the annotation process and reduce the time to prepare annotated data for machine learning algorithms. Thus, the model receives only partial information about the performed activity. Therefore, we propose an LSTM-based architecture, which takes into account both the ragged tensors and the weak labels. To the best of our knowledge, no prior studies attempted counting utilizing variable-size IMU acceleration data with relatively low computational requirements using the number of completed repetitions of hand-performed activities as a label. Hence, we present the data segmentation method we employed and the model architecture that we implemented to show the effectiveness of our approach. Our results are evaluated using the Skoda public dataset for Human activity recognition (HAR) and demonstrate a repetition error of ±1 even in the most challenging cases. The findings of this study have applications and can be beneficial for various fields, including healthcare, sports and fitness, human–computer interaction, robotics, and the manufacturing industry.

## 1. Introduction

In recent times, people use more and more new technologies, devices, and sensors that generate data to support their daily activities. Researchers can use this sensor data to identify the human body’s actions and movements for human activity recognition, or HAR, as it is more commonly known. Various sensor types collect data in those settings, such as the ones that use video and inertial measurement units (IMUs). Sensors provide means to capture data related to human activities, which can be used to develop machine learning models for human activity recognition (HAR) and human behavior recognition (HBR). Achievements have been made in sports and entertainment [1,2,3], industrial applications [4], and healthcare [5]. Meanwhile, the academic community is actively researching innovative sensor technologies for human activity and behavior recognition, including new sensor designs, applications of traditional sensors, and the usage of non-traditional sensor types [6]. Many studies are currently being conducted to improve existing approaches or solve newly identified problems for the detection and classification of activities with supervised or unsupervised techniques. The majority of research studies and applications in the field of HAR have, up to this point, focused on detecting activities, such as walking, standing, and sitting, as well as other daily living (DL) activities [7], and analyzing their characteristics to generate new insights [8].

Building upon this existing body of work, the scope of our study is to highlight the counting of events that occur in a given period in different activities of daily human life (DHL) or daily work life (DWL). We focus on counting the end of an activity to determine the number of times that activity occurs. By centering our attention on event counting, we aim to provide a comprehensive understanding of the frequency and occurrence of specific actions within the broader context of human activities. In daily activities, such as workouts or sports, it is critical to correctly segment and recognize the type of activity using a sophisticated model [9]; however, mainly classification models can offer such information. Besides that, it is important to acknowledge that different fields exhibit variations in sensor types, signal characteristics, produced by these sensors and face different challenges. With small adjustments, counting with AI and IMU data can be used in the industry to solve a variety of problems. Some examples include: (i) Sensor data analysis to monitor the performance of equipment, detect anomalies, and optimize operations; (ii) Quality control to count the number of defects or errors and improve the quality of products and reduce costs; (iii) Safety monitoring to count the number of incidents to improve safety and reduce the risk of accidents in industrial environments. For example, in an industrial setting where the tasks are more complicated, workers have many repetitive tasks to complete daily, such as screwing activities during assembly processes, which they occasionally miscount or forget to execute [10]. In this regard, we aim to provide people with information and raise awareness about the number of completed activities.

As Kim et al. [11] stated in their work, counting is one ability that humans usually acquire from a young age, and while it appears to be a simple task, young people still need a long period to master it. Comparably, it is challenging to develop a model that can count the number of completed activities (CA) in a time period, based on data from Inertial Measurement Units (IMUs) or similar body-worn sensors. With the term “completed activities”, we refer to the repetitive activities that can constitute a single work step in a workflow, e.g., the screwing of one screw, which is complete with the tightening of it.

The analyzed data for our research are sequences of varying length annotated with weak labels that serve as targets for the machine learning models. Traditional approaches for handling time-series data often involve dividing the data into fixed window lengths. However, due to the high variance in activity durations, using a small window for a long activity or a large window for a short activity can result in the loss of important information [12]. Up until recently, one commonly used approach required very large window samples that could fit all activity sizes inside, padding them with a value (typically zeros) and feeding them as input to the networks. Annotating data, on the other hand, is usually laborious and time-consuming, and requires considerable attention and precision.

As an overview of the challenges motivating this work, we concentrate on the following: (i) spread of valuable information across consecutive sequences, (ii) information loss caused by using a single, fixed window size for varying-duration activities, (iii) limited flexibility of models that are more specific for particular data due to manual preprocessing methods, and (iv) the topic of data annotation. To address the aforementioned issues, we propose a model design that works with (i) variable length of data as input, (ii) data that have some form of annotation but is not completely annotated, known as weakly labeled, (iii) raw calibrated data that are normalized but not subjected to any further filtering, to reduce complexity, simplify the preprocessing stage, and develop a more robust model in unprocessed data to achieve counting, (iv) the implemented counting method, integrated within the model’s training process rather than simply incrementing the count of correctly classified instances, and (v) fewer training parameters than existing model architectures in the literature to enable suitability for deployment on devices that have limited processing and power resources. Our approach aims to improve the model’s ability to accurately count the activities performed by a user, rather than just detect them. To the best of our knowledge, this is the first study that investigates the counting of completed activities and tasks, in a way that goes beyond counting the quantity of previously correctly recognized activities from a classifier, employing an LSTM [13] for counting patterns in a sequence.

Counting is an essential skill that humans employ in their daily lives across a wide range of activities and tasks. Whether it is a simple task or a more complex one, the ability to accurately count holds immense value and can benefit from technological assistance in various fields. According to our literature review, studies in this area mostly used video-capturing sensors and have been conducted in the sports or medical sector.

Fang et al. [14], in their study, explored the possibility of counting the number of items in a display and raise the question, “Can a recurrent neural network learn to count things?”, with their findings favoring a positive answer. While they also used an LSTM model, our model takes as input unfiltered accelerometer data relating to human activities in daily life. In a different setting, the authors of [15] proposed to count repetitive activities in a video by sight and sound using an audiovisual model, which differs from our approach among others in the choice of the sensors, since we aim to use body-worn sensors. In ref. [16], the MM-Fit dataset is introduced, which contains data from inertial sensors and ambient video sensors capturing full-body workouts. A single 3D accelerometer worn at the chest is employed in [17] to recognize four types of workouts and count repetitions after the workout is firstly determined and classified by their algorithm. Another study focused on fitness exercises is the one by Ferreira et al. [18], where the authors select camera sensors for realizing their approach to do workout repetition counting. The researchers in [19] designed and implemented a body capacitance-based sensor and employed a residual deep convolutional network that uses dilated convolutions for recognizing and counting gym workouts, while their approach had competitively high counting accuracy, we opt for sensors available in devices of daily use, such as smartwatches or smartphones, and utilize unfiltered data in our approach. In [20], with 91% of the used Cross-fit exercises having an error within a margin of ±1 repetition, the authors used a vibration signal during their data collection and trained a neural network for counting that relied on whether an input window contains a repetition start. However, our model uses only weak labels as target data for its variable-size input, which requires less human annotation effort than models with dense labels.

Weakly labeled data can be beneficial for deep learning algorithms in certain situations and refer to data that are only partially labeled, meaning that they have some form of annotation, but not all the information is present. This type of data is less expensive and time-consuming to obtain than fully labeled data, and it can be used to train deep learning models in a semi-supervised manner. The authors in [21] proposed an attention-based convolution neural network to process weakly labeled human activities and recognize them. The dataset contains information only about the type of activity that occurred in a sequence of sensor data. A weakly labeled dataset was also included in a Dual Attention Network For Multimodal Human Activity Recognition Using Wearable Sensors in [22], where they blend channel attention and temporal attention on a CNN, for multimodal HAR. The activities that are contained in the dataset are walking, jogging, jumping, going upstairs, or going downstairs, and have a significant difference from the activities that we explore and the way that we create our training dataset. In a related field, for locomotion activities, several studies explored step counting using IMUs or smartphones [23], including approaches that utilize deep learning techniques. One such approach is the attention-based LSTM model by the authors of [24], which has been shown to effectively count steps with high accuracy. However, unlike the continuous and repetitive movements associated with step counting, our approach concentrates on hand-performed activities that involve discrete movements. Furthermore, our model maintains simplicity regarding processing resources, low power consumption, and suitability for edge computing devices.

Raw data as input for the models have the advantage of reducing the need for pre-processing techniques, which can be a time-consuming and resource-intensive task. In our study, we refer to the public dataset’s raw calibrated data that have not been subjected to any further preprocessing steps other than normalization. When working with raw data, the model can automatically learn useful features from the data, which can save computational resources and reduce the risk of human error. Important contributions have been made by Shen et al. in [25], where they proposed a workout tracking system that uses smartwatches to accurately and efficiently track both cardio and weightlifting workouts without the need for user input. Their counting strategy begins with detecting and labeling weightlifting sessions, followed by a naive peak detection algorithm based on auto-correlation results. They filter out non-repeating signals and calculate the number of repetitions by counting detected peaks. Likewise, Prabhu et al. in [26] also based their approach on classifying the activities before counting with a peak detector method. Their research aims to identify the most effective artificial intelligence model for repetition counting in LME exercises to be used in wrist-worn rehabilitation programs.

In their work, Taborri et al. [27] implemented the following algorithms, one for recognizing activities based on SVMs and one for counting actions related to workers in the industry. Twenty-three body-worn sensors collected data from the participants, which were divided into windows of 0.6 s and had features such as mean, standard deviation, maximum, and minimum, were computed for each activity. Physical exercises for indoor and outdoor environments were used to recognize the real-time segmentation and classification algorithm in [28]. The method they proposed requires one sample of data for each target exercise; however, once more, the counting relies on accurate classification of the activities. Another algorithm in the context of human activity recognition that segments repetitive motion is the one presented by the authors in [29]. This algorithm was utilized to identify similar location patterns in indoor localization and addresses the problem of subsequence search in univariate and multivariate time series. An automated segmentation way and labeling of single-channel or multimodal biosignal data using a self-similarity matrix (SSM), generated with the feature-based representation of the signals, is proposed by the authors in [30]. Examples of data with the few-shot learning were employed by Nishino et al. [31] to recognize workouts using a wearable sensor including data augmentation and diversification techniques for their data to achieve repetition counting.

In our approach, we leverage deep learning models to extract features and train them to accurately count activities using public datasets that contain raw and calibrated data of human activities performed with hands. As with many machine learning techniques, we normalize the raw calibrated acceleration data before we feed them to the deep learning model. However, we do not apply additional preprocessing steps or filtering to the data. Moreover, the sensor placement described by the dataset’s authors in [32] is essential to recreating the study’s results. To train the model, we divide the data into segments of variable sizes with weak labels that utilize only the number of repetitions of activities for each sequence as the target value. Hence, the model learns to count activities regardless of the sequences’ size, which is important for real-world applications, where activity durations may vary.

## 2. Materials and Methods

### 2.1. Counting Approach

Counting repetitions in a sequence is a fundamental problem in various fields, such as speech and image processing, bioinformatics, and cognitive science. Many methods for counting can be deployed, the majority of which require hand-crafted rules, feature extraction and statistical methods, or rule-based systems to manually count objects or events. As was described previously, neural-network-based approaches can be used to count repetitions in a sequence with a combination of CNNs and RNNs or encoder-decoder architectures, trained on a labeled dataset of sequences, to learn a mapping between the input sequence and the number of repetitions. Focusing on a system that can handle weakly labeled data while being less reliant on human intervention and more automated can reduce the complexity of the counting process, make the model more robust, and provide flexibility for applying the method to a wide range of problems.

Weakly labeled data offers a cost-effective and efficient alternative to acquiring fully labeled data, as it is less expensive, time-consuming, and tedious. This type of data enables the use of semi-supervised learning approaches, which can be beneficial when considering the annotation cost associated with large and complex datasets. By leveraging weak labels, the model is encouraged to learn more generalized patterns in the data, leading to improved performance on unseen examples. For our experimental setup, we use IMU acceleration data from daily human activities performed at a quality control checkpoint in a car maintenance scenario that captures activities relevant to the inspection process. Thus, it provides a representation of real-world conditions by addressing the complexity of variable-length data, to develop robust and realistic models for activity counting and recognition tasks.

Despite the fact that counting repetitions in a sequence with variable-length data is more challenging than counting repetitions in a sequence with fixed-length data, this structure is more realistic because data from signals, time series, texts, and other sources have varying length. Using fixed-length tensors for the data can be efficient in certain situations because they simplify the problem, as the model only needs to process a fixed amount of data, regardless of the length of the input sequence. Furthermore, because the libraries and software tools required to build the model are more widely accessible, its implementation and deployment may be simpler. However, using variable length tensors can also be beneficial in many situations. They allow the model to handle input sequences of different length, which is important when dealing with complex real-world data and activities of various length. Additionally, variable-length tensors enable the model to process the entire input sequence at once, rather than only a fixed-length subset of it, which can be valuable when the position of the repetitions is not known in advance.

In our approach for counting, we use data from public datasets that contain data from human activities in the car manufacturing industry, recorded with IMU sensors. We create sequences of data that have a variable size and we obtain a weak label for each sequence. The label shows the executions number of one type of activity observed in the sequences, which is fed into an LSTM regression model built with the ragged tensors. For each sequence that is input to our algorithm, one single count is predicted as the output.

### 2.2. Dataset

**Figure 1:**
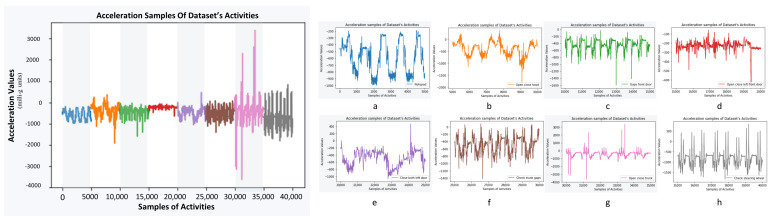
This figure shows examples of the signals that represent each class of the Skoda Public dataset with different colors. Starting from left to right (**a**) Notepad, (**b**) Open close hood, (**c**) Gaps front door, (**d**) Open close left door, (**e**) Close both left door, (**f**) Check trunk gaps, (**g**) Open close trunk, (**h**) Steering wheel. For all the activities, samples are taken from the X-axis accelerometer on the right hand. The activities of the “open hood” and “close hood” as well as the “open left door” and “close left door” are displayed together as “open close hood” and “open close left door”, since they are always consecutive. The acceleration is provided in milli-g units.

The data used for this study are part of the Skoda Public dataset [32], which includes repetitive activities regarded as single, discrete actions as opposed to continuous activities, such as walking or running. The example signals of the manipulative gestures of the dataset that were performed in a car maintenance scenario, visualized in Figure 1, are “write on notepad”, “open hood”, “close hood”, “check gaps on the front door”, “open left front door”, “close left front door”, “close both left door”, “check trunk gaps”, “open/close trunk”, and “check steering wheel”. These activities were recorded for about 3 h by 20 sensors placed on one subject’s left and right upper and lower arms. For each sensor, there are acceleration values on the x, y, and z axes that are calibrated in milli-g units (1000 = earth gravity vector, which in S.I. units would be 0.001 g or 0.00981 m/s${}^{2}$), and the sensor sample rate is approximately 98 Hz, as stated by the dataset’s authors.

**Figure 2:**
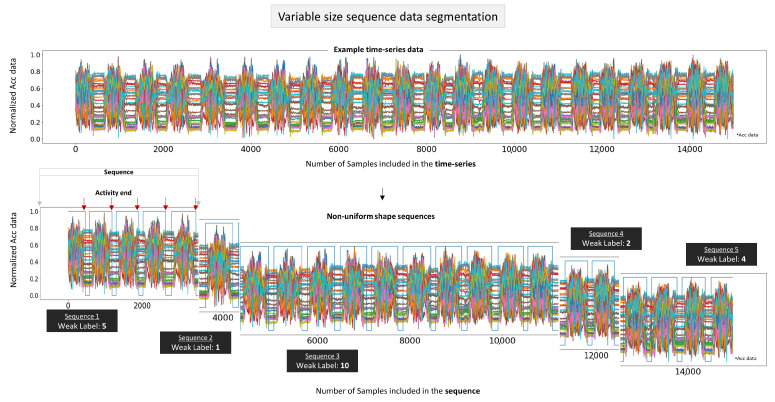
This figure presents the non-uniform shape input for the neural network. We visualize on top of the image time-series data that will be divided into five distinct example sequences of segmented acceleration data with varying duration, number of samples, and weak labels that have a range from 1 to 10 counted activities. The blue squared line shows the start and end of each activity within each sequence. The weak label is generated by the number of spotted endings (red arrow) inside each individual sequence.

The objective of this work is to count how many times one activity happened in a period of time, e.g., detect in the data patterns how many times the person closed the hood in the activity “close engine hood”. The dataset contains a dense label for each sample, which allows the detection of the end of the activity. The weak label targets are generated by “recording” one repetition for each task completion. Therefore, for the training phase, for every instance where a task is successfully completed, a single repetition sample is marked as “activity end”. By using this approach, we create weak annotations that indicate the presence of completed repetitions, allowing the model to learn and recognize the patterns associated with activity completion. We normalize the data with minmaxscaler [33] in a range of [0, 1] and divide it into variable-size sequences. By using an algorithm to generate an array of random numbers, we split the entire dataset into segments, which define the sample length of the sequences, as shown in Figure 2. For example, if we want to generate 20 sequences of variable-length data, the algorithm will create 20 random numbers between 0 and the dataset’s maximum index value. Next, we replicate the original data for each activity to provide our model with a larger dataset to train, without using data augmentation techniques for generating variation in the signal’s patterns. This expanded dataset introduces greater variability in the unique length of activity sequences and the number of activities contained within them, thereby enhancing the model’s robustness.

The labels for each sequence in this dataset are produced by the number of spotted endings or finished tasks in the sequences, where the last timestamp of each observed activity adds 1 count to the final label of each unique sequence. Figure 2 visualizes the division of a time series into sequences of variable size and how the weak labels are formed. The term “weak labels” in our method denotes the absence of data annotations that map the start and end of an event in the sequence. The number of activities in the sequence is the only information of the sequence that the model utilizes as a target value.

We divided the subsets of activities into 600–900 sequences, where 100 of each type were left as a test dataset, as presented in Table 1, and 10% of each training dataset was used as a validation set. Consecutive activities, such as “open left front door” and “close left front door”, were merged into one class, as explained in Figure 1. In this case, the algorithm must count +1 when one of the activities of interest is happening. In the last entry of the table with the label “combined activities”, one can see results with 8000 training sequences, for a class that is generated with combined data from all the previous classes in a single one. Every activity that is not a null class will be counted in this scenario, to distinguish between an occurring activity and null class without considering the type of activity. We created 8000 varying-length sequences from all classes, of which 7000 were used to train the network. Despite the more complex approach, employing variable-size sequences allows us to extract the most valuable information from our data without padding.

### 2.3. Counting Algorithm

A fixed window approach is a commonly used method for segmenting time-series data before using them as input to deep learning models. The segmentation is based on characteristics of the event that we want to identify, such as its periodicity, frequency, and length, among others. Despite their ease of implementation and interpolation with other libraries, fixed-length tensors with a predefined shape have limitations. For example, they are not well suited to handling non-uniform shape data, such as sequences of varying length, without the need for padding or truncation, which can result in additional noise to the data, increase in the computation time, information loss, and storage inefficiency, and it may not always be appropriate. To address the above issues, we used TensorFlow’s ragged tensors [34], which support variable-length sequences of samples.

**Figure 3:**
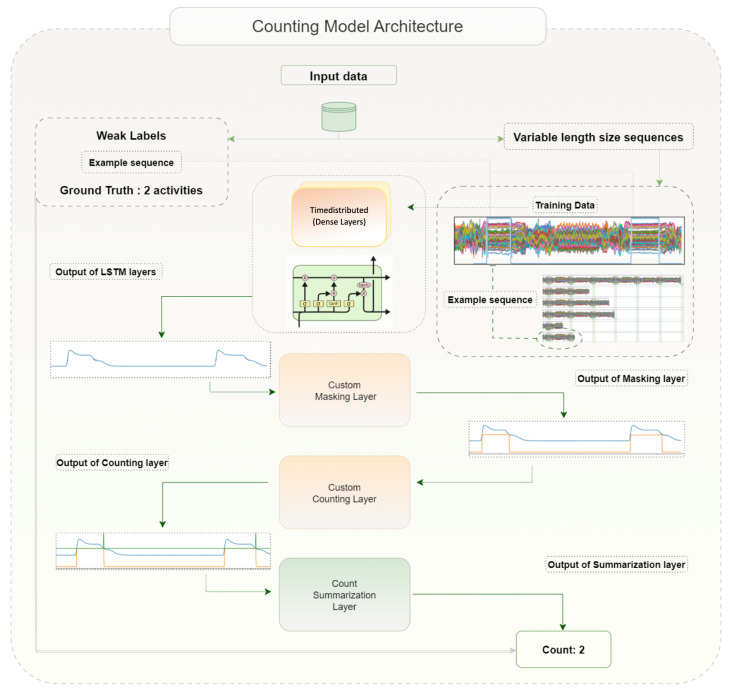
This figure presents an example of the model architecture that was used for the counting of activities. The acceleration data are divided into variable-length sequences and then used as input to the model. For each sequence, there is one weak label that is generated by the number of activities that are included in the sequence. Two time-distributed dense layers process each sensor reading independently before entering an LSTM layer where we get an output for each time step. Since the input data have a variable size, ragged tensors are employed for this task. The output of the LSTM part is inserted into a mask layer that detects values above a threshold and converts the signal into a square form before it continues to a layer that detects the created “edges” of the square shape and gives a final summation of all edges of the sequence to one single number.

In this study, nine sub-datasets were used with our algorithm to count activities, with nine separate trainings for each subset. Figure 3 shows the architecture of the model that is used for the counting task. The acceleration data are separated into variable-length sequences, each of which comprises several activities and is used as input data, as was previously mentioned. The weak label that the model uses as target data is the total number of activities in each sequence. The model learns to relate acceleration data to the number of activities, so when we feed as input “ new unseen” acceleration data of variable length, it outputs the number of spotted activities. The annotation provided no information about the location of activities within the sequence, nor does it provide any additional supporting details to guide the model. A large grid search was deployed to explore the best combination of parameters for the number of layers, learning rate, batch size, optimizer, loss function, and activation functions to use in our network. We experimented with various hyperparameters to achieve the best performance. For the number of layers, we tried configurations ranging from one to three time-distributed layers and one to 4 Lstm layers. For the learning rate, we tested values such as 0.01, 0.001, 0.0001, and 0.00001. We also explored different batch sizes, including 2, 4, 8, 16, 32, and 64. As for the optimizer, we experimented with Adam, RMSprop, and SGD. We evaluated loss functions, such as mean squared error, mean absolute error, and Huber loss. Finally, we tried different activation functions, such as ReLU, tanh, and sigmoid, to achieve the optimal performance on our task.

**Figure 4:**
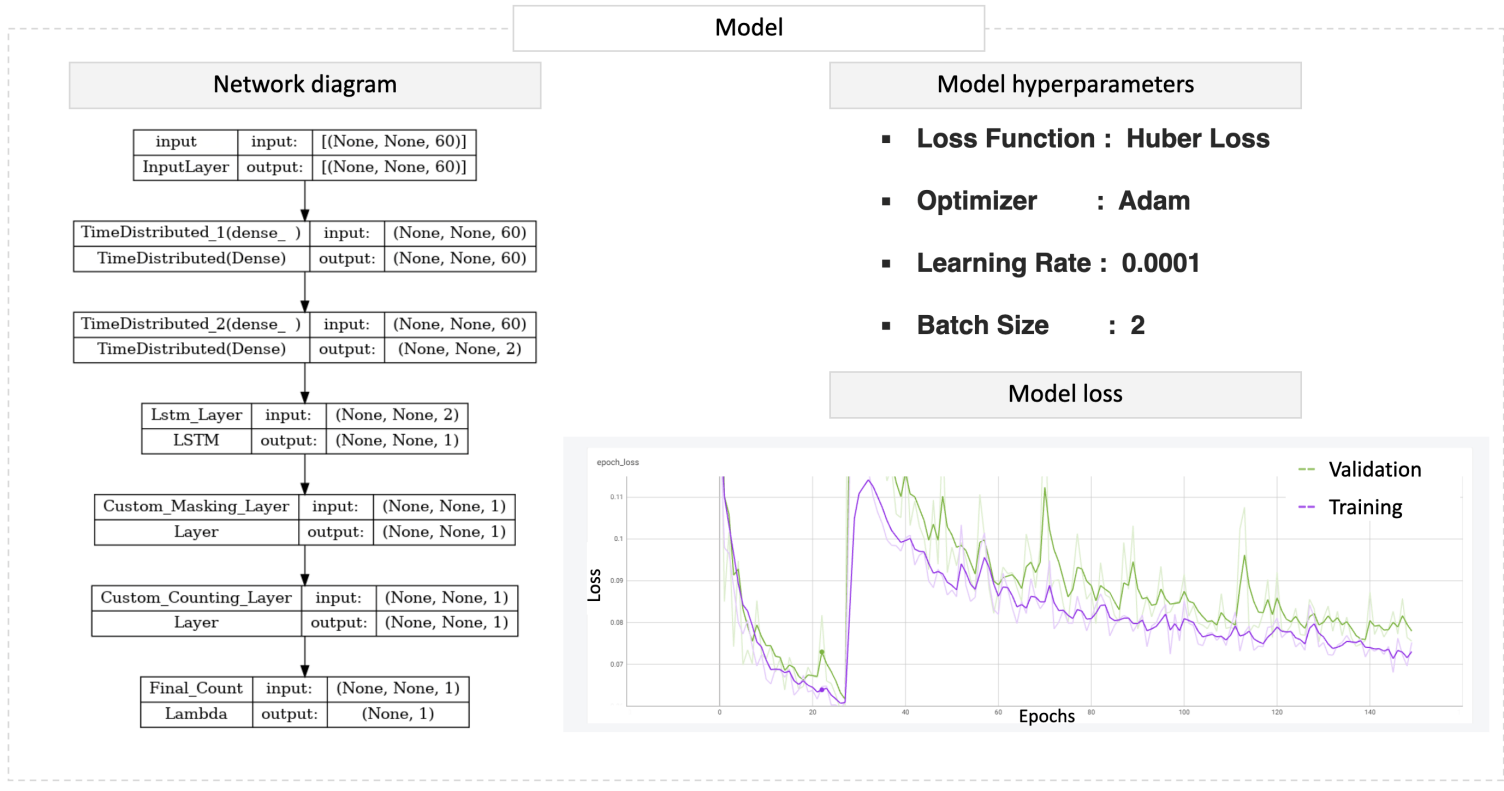
From left to right, this image presents the network’s diagram of the counting model with the input (60 acceleration signals) of variable length and output of 1 number. Moreover, the hyperparameters include Huber loss, ADAM optimizer, a learning rate of 0.0001, and a batch size of 2. An example of a learning curve for the training and validation sets demonstrates the model’s performance during training. The *x*-axis represents the number of training epochs, while the *y*-axis represents the loss metric.

As shown in Figure 4, two dense layers were used at the beginning of the model to reduce the dimensions of the input data before entering the LSTM. The first dense layer is composed of 60 neurons (number of input signals), and the second consists of 2 neurons with the rectified linear unit (RELU) as an activation function. Two custom layers are then placed after an LSTM layer that outputs a three-dimensional sequence and has one neuron with a linear activation function. The custom masking layer thresholds the signal and converts the output of the LSTM to a more binary format, and xthen, the counting layer counts the regions where the signal value is not zero and summarizes them to one final number, as shown in Figure 3, of the output’s graph. After experimenting with several parameter values as mentioned above, a batch size of 2 with a learning rate of 0.0001 and the “Adam” optimizer were selected for optimizing the model. The algorithm’s performance is evaluated using the Huber loss as the loss function, which is a combination of the mean squared error (MSE) loss function and the mean absolute error (MAE) loss function. This combination improves the performance of the model when outliers are present in the data, which is possible in our study because the input sequences were generated arbitrarily.

## 3. Results

In our study, we applied deep learning approaches to acceleration data to count the number of activities in variable-length sequences, as presented in the model architecture.

The ground truth in Figure 3 is two activities in the illustrated example sequence. It is evident that the LSTM outputs a signal with two peaks, which is then converted to a binary format by the masking layer, and we count +1 at the edge of each square area. Two is the final result predicted by the model for the specific input sequence. Figure 4 visualizes the learning curve of the training and a validation loss to present the model’s performance during the training of the “notepad” dataset. After each training, we evaluated the model with unseen data sequences of the same class, and the results show that the model can predict very close to the weak label.

**Figure 5:**
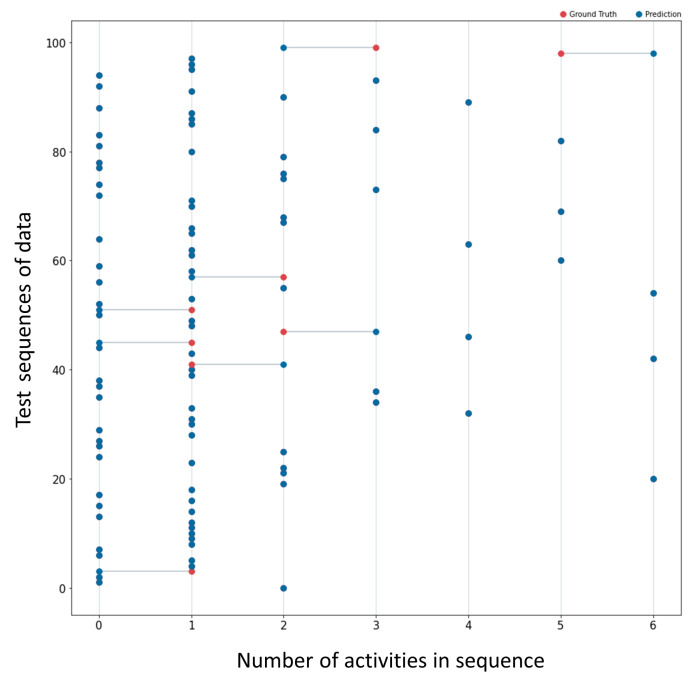
The figure presents the ground truth (red dots) and prediction (blue dots) of the model for 100 unseen sequences of data. The input data are from the notepad writing activity of the Skoda dataset. The algorithm for counting predicted accurately 92 out of 100 activities. A line connecting the two numbers shows the difference in the incorrectly predicted sequences. The largest error per sequence observed in the graph is 1 count.

**Figure 6:**
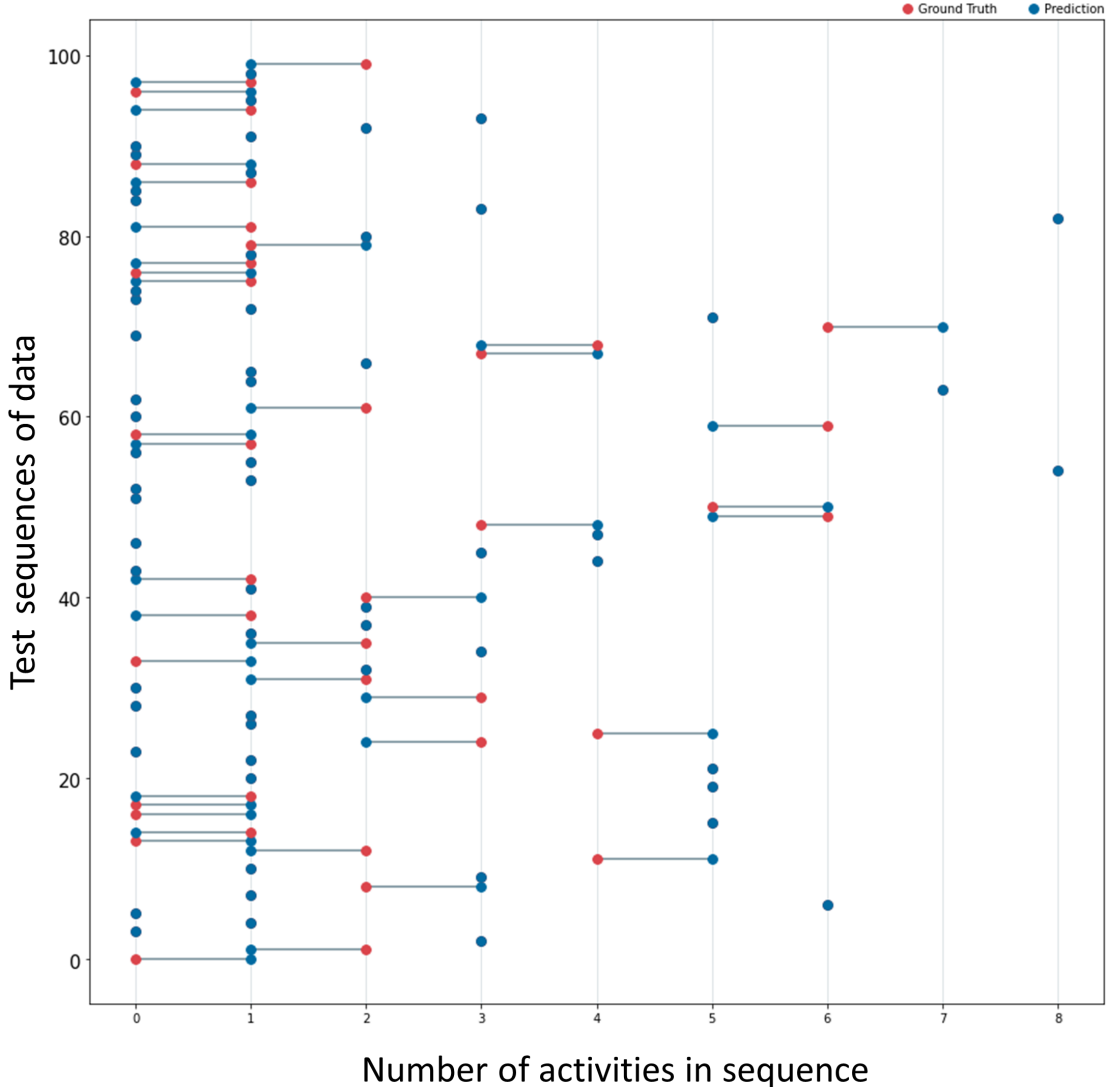
The figure presents the ground truth (red dots) and prediction (blue dots) of the model for 100 unseen sequences of data. The input data are from the steering wheel activity of the Skoda dataset. The algorithm for counting predicted accurately 60 out of 100 activities. A line connecting the two numbers shows the difference in the incorrectly predicted sequences. The largest error per sequence observed in the graph is 1 count.

For two of the activities of the Skoda Dataset, we visualize example results of the weakest and best cases of the model’s predictions in variable-length data sequences. In Figure 5, one can see the graph showing the ground truth and prediction of the model for the activity “writing in notepad”. There, we trained the model with 500 sequences of variable size and variety in the number range of contained activities. The findings indicate that the model predicts 92 out of 100 correctly, while the remaining 8 predictions have an error of one activity. Similarly, even though the model predicted less accurately for the dataset’s “steering wheel” class, the predictions have a maximum error of one activity, as shown in Figure 6. Table 1 contains information regarding the training data and the results of all dataset activities. The discussion section provides further details about the results.

The table shows in approximation the number of data samples contained in the original dataset for each class, the number of training and test sequences, the range of the number of activities included in different sequences, the accuracy in the test data, the mean absolute error, and the mean % accuracy of test sequences in the test data.

The “notepad” class has the smallest MAE, 0.08, and the highest mean % accuracy, while the “steering wheel” class has the highest, 0.4, and the lowest mean % accuracy. In the “combined activities” class, the algorithm counts interesting activities in a sequence, regardless of the activity type, in a dataset consisting of all classes combined into a single one. The table lists 8000 training sequences of varying length, of which 7000 were used for the network’s training. Randomly, 1000 sequences were kept as test data, and the number of activities in 765 out of 1000 was predicted correctly. From those 1000 sequences, 230 had an error of ±1 counts, 3 of them an error of 2 counts, and 2 of them an error of 3 counts. As shown in the table, the mean accuracy is presented as a percentage, representing the average accuracy of 100 sequences from each test dataset. For example, for the “open close trunk” dataset, the mean accuracy for the 100 test sequences is 81.65%. This means that we found the accuracy of the model for each predicted sequence of this test dataset, and subsequently, provide an average estimate of the accuracy across all the test sequences to evaluate the performance of the model. The dataset accuracy evaluates the model’s ability to predict the exact number of repetitions accurately, while the mean accuracy gives an overall measure of the model’s performance by indicating the average deviation of the predicted number of repetitions from the true value across all test sequences.

## 4. Discussion

The current study confirms that it can count interesting events in time series with more flexibility concerning the size of each input sequence from a model that uses (i) solely normalized data, (ii) weak labels, and (iii) deep learning. According to our method’s preliminary findings, when we train the model for specific activities, our algorithm can accurately predict, in most cases, the number of times an event is repeated in a sequence. For some of the activities, the prediction is better than others. For example, activities such as “open close hood” and “open close left door” contain patterns of both opening and closing the object, which can possibly create a larger confusion for the model to recognize the pattern. The lower results were achieved for the steering wheel class. The wheel rotates three times in each direction, clockwise and counterclockwise, before switching. In this case, the orientation for each side within the same sequence may be contributing to the confusion, or the data may not be sufficient for the model’s design and a deeper architecture or new data may be needed to capture the dependencies. Likewise, for the “combined activities” class, in each sequence, the model may contain patterns from one or more different activities that need to be counted. However, due to the weak labels, no other information about the activity type is available, except for the total number of events contained in each. The consistently small error, typically within the range of ±1, demonstrates the effectiveness of our current architecture in accurately counting activities. However, it also highlights the potential for further research and improvements to reduce this error even further and achieve even more precise activity counting results.

The model must be trained on a dataset of labeled sequences where the number of repetitions in each sequence is known, regardless of the architecture being used. To address the diversity in the different activities without padding and by utilizing the entire information of the sequence, weak labels that are less time consuming and variable-size sequences are used. Nevertheless, it is important to note that using weakly labeled data does introduce certain limitations since the data are only partially labeled. A more comprehensive target for the model might be provided, for example, by a second model trained with information on the type of activity occurring, the location, the duration, or even data examples to use for training. In our case, we developed our ragged tensor model using calibrated data, that we normalized on a range of [0, 1] to ensure a common scale. We selected the calibrated version because it is in S.I. units and can be replicated by anyone even though the raw data of the public dataset with our model provided comparable results. Additionally, the calibrated data support the use of any sensor that takes readings using the same units, not just the specific sensor that the dataset’s authors used in their study. Despite the benefits of ragged tensors, such as efficient storage and easy handling of variable-length data, working with them proved to be challenging, requiring additional effort and consideration of alternative approaches, as some operations, libraries, and software tools outside of the TensorFlow environment are not currently sufficiently supported.

Counting with deep learning can be beneficial for a variety of fields, such as health care, to monitor and track physical activity levels and rehabilitation progress, sports and fitness, to track and analyze athletic performance, robotics applications, to detect and track human movements for use in collaborative robots, identifying actions and events in industrial settings, etc. These are only a few examples of how counting with AI can be used for the analysis and interpretation of IMU data in industrial applications. Although depending on the problem that needs to be addressed and the kind of data that are accessible, a specific application must be selected and modifications and optimizations must be completed. Furthermore, merging AI with technologies such as edge computing, IoT, and cloud computing for data analysis in real time and making decisions based on the results can improve these applications. Initial contributions in this direction have been made in [35], where the authors discuss the transition to real-time models, as well as in [36], where the authors introduce their system that uses wearable sensors to capture online data and perform activity recognition using Hidden Markov Models.

Our next steps include the improvement of the current model to count activities from data that are not collected in laboratory settings to enable better generalization and make it more robust across various sources of IMU data. Counting different activities within one sequence would be another challenge to address. Besides that, it is interesting to investigate the potential application of our method to other time-series data. However, it is important to consider individual differences in movement patterns that may be influenced by factors such as body size, gender, and age. One approach is to use a diverse dataset of individuals with varying body sizes, genders, and ages to train a deep-learning model that can generalize to new individuals and accurately estimate their movements. Additionally, optimizing the placement of the sensors can improve the accuracy of the model and reduce the need for unnecessary sensor data, which can be an obstacle in the learning process.

## 5. Conclusions

In this paper, we presented a counting method for activities and tasks to identify the end of an activity based on raw calibrated acceleration data that are weakly labeled. The generated sequences from the data have variable sizes instead of a fixed window size, which restricts the system to operate with specific settings and makes it more challenging to fit different types of activities with varying duration. An LSTM model for regression analysis was developed for the task where we tested its performance with data from different classes of the Skoda dataset for HAR. Our research focuses on using raw calibrated data rather than preprocessed or filtered data to build models that are more resistant to changes in their settings and generalize better to different scenarios. Furthermore, when fully labeled data are not available, the use of weak labels reduces the expense of data annotation.

Our results indicate that our method can count instances of activities when it corresponds to a single type and has shown promising results when training input data contain multiple types of activities. This is supported by the findings in the aforementioned cases, which state that the error for the sequences was always within ±1 iteration. Our next stages will involve testing the model using more complex data collected under real-world conditions and making it more robust against outliers and different sources of data. Thorough investigation and application of this method in other domains, such as object detection in videos, can also be included in the scope of the counting problem while adapting the approach to work with the minimum required sensor signals to produce accurate results to create user-friendly solutions that can be used during the daily work life.

## Figures and Tables

**Table 1:** This table lists the overall summary results for accuracy and MAE across all activity classes for the test datasets. As one can see, it contains nine separate datasets of activities. For each activity, the samples of the original dataset, the variable length training sequences created from the data, the range of the number of activities within the sequences, the test sequences, and the results such as the dataset accuracy and mean accuracy in 100 sequences of each test dataset are available. The counts’ range shows the maximum number of activity counts contained in 100 sequences of different lengths of the test dataset. The mean percentage accuracy provides an overall assessment of the model’s performance by displaying the average deviation of the predicted number of repetitions from the true value across all test sequences, whereas the dataset accuracy assesses the model’s ability to predict the precise number of repetitions accurately. The last entry in the table represents a class where all the data from all classes were combined into one class and then split into variable-length sequences so that the model is trained on more complex data. In that case, the model learns a larger variety of patterns from all classes as a single activity class and must identify between the activity class and the null class to perform the counting.

Training A/A	Activity	No of Samples in Original Dataset	Training Seq.	Range Counts in Test Seq.	Test Seq.	Test Dataset Accuracy	MAE	Mean % Accuracy in Test Seq.
1	Steering wheel	51,904	500	0–08	100	60/100	0.4	72.19
2	Check trunk gaps	70,000	500	0–07	100	89/100	0.11	91.66
3	Notepad	74,000	500	0–06	100	92/100	0.08	96.12
4	Open close hood	186,399	800	0–07	100	70/100	0.3	78.58
5	Open close left door	82,000	600	0–12	100	68/100	0.33	80.22
6	Gaps front door	60,000	500	0–09	100	84/100	0.18	90.38
7	Close both left door	72,000	500	0–06	100	75/100	0.25	79.22
8	Open close trunk	95,000	600	0–10	100	74/100	0.26	81.65
9	Combined activities	705,904	7000	0–11	1000	765/1000	0.242	81.29

## Data Availability

Skoda public dataset for Human activity recognition.
